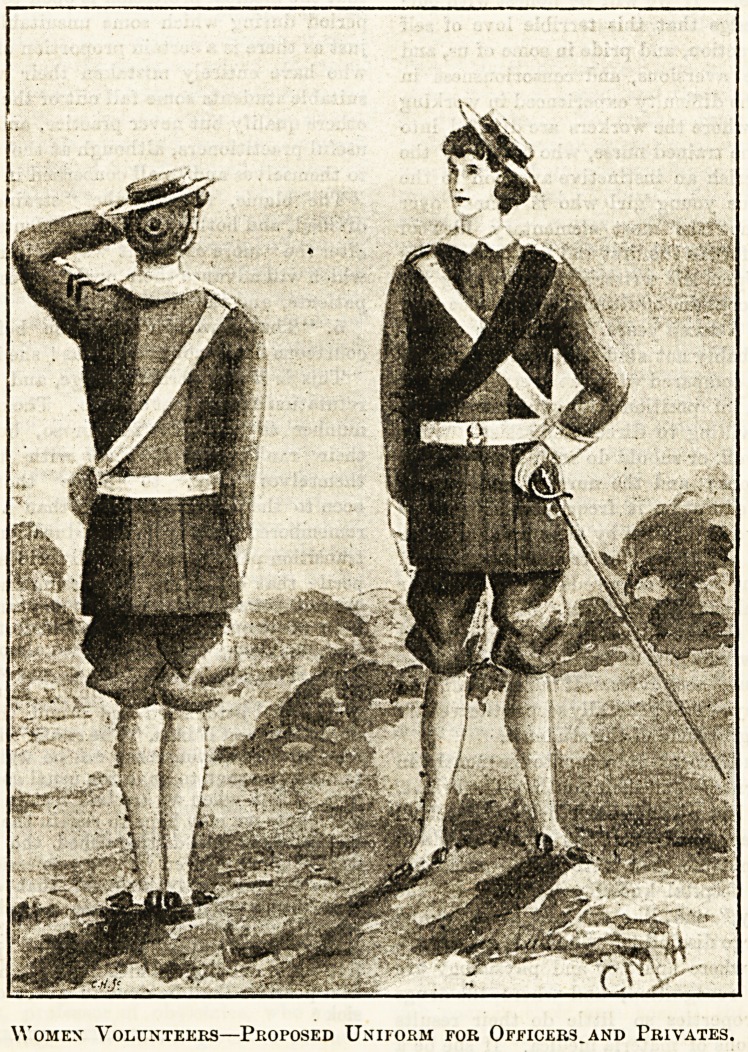# The Hospital Nursing Supplement

**Published:** 1894-04-21

**Authors:** 


### "The Hospital" Nursing Mirror.

Being the Extra Nursing Supplement of "The Hospital" Newspaper.

[Contributions for this Supplement should be addressed to the Editor, The Hospital, 428, Strand, London, W.C., and should have the word "Nursing" plainly written in left-hand top corner of the envelope.]

### News from the Nursing World.

### "THE HOSPITAL" CONVALESCENT FUND.

We are glad to be able to report that our readers have for some time now, helped us to help others, by contributing towards a "convalescent bed" in connection with an instititution for the reception of weary workers recovering from illness, or in great need of rest and change. Useful as such a scheme must certainly be, some of our widely-scattered readers have pointed out that our plan of fixing on one spot had drawbacks. The journey was long for nurses in distant parts of the country and the expenses of travelling proportionately large. The managers of the Fund are, therefore, arranging that in future nurses shall select their own locality, as far as possible, the Fund paying the whole or part of the expenses, according to the nature of the case. The most urgent will receive first consideration, and applicants must show that they are not able to secure what they need without the help of the Fund, and they should address the Hon. Sec., The Hospital Convalescent Fund for Nurses, 428, Strand. Contributions should also be sent to the same address, and they will be acknowledged in the columns of the Nursing Supplement, where also a report of the progress of the Fund will appear from time to time. Our readers are reminded that this Fund is entirely dependent on their generosity and sympathy for its existence, and the extent of the help afforded to weary nurses is only limited by the amount of their donations. It is therefore hoped that subscribers will become more numerous, and that all will realise that time and trouble will not be grudged to promote so good an object as the one in question.

### A MUSICAL TREAT.

A capital concert was enjoyed by the nursing staff of St. George's Hospital on 5th inst., and was repeated on 6th to enable all to participate in the pleasure provided for them by the resident medical staff. The musical programme was varied and excellent, and the Board room was well filled. At the close of this most successful entertainment a cordial vote of thanks was offered to the ladies and gentlemen who had so kindly given their services.

### UNCERTAIN QUANTITIES.

In a recent lecture on "Recreation for Nurses," the present matron of St. Bartholomew's Hospital is reported to have said that "occasional hours off duty" during the week fall to the lot of her nursing staff. The "regular" probationers' privileges in this respect are apparently too unimportant to be mentioned, for they are markedly omitted from prospectuses sent out to candidates. The paying probationers are more fortunate in receiving printed information that "occasional hours" in their case mean two hours daily.

### USEFUL LESSONS.

In Dr. Sansom's appeal for the North-Eastern Children's Hospital at the meeting reported last week, he spoke of the little hospital as not only a place for tending the sick, but also as a civilising institution for the locality in which it exists. As each little patient is the centre of interest to a nnmber of its friends during its residence in hospital, the "civilising" process possibly extends in some cases over a vast area, and the little patients are not only benefited themselves by intelligent treatment, but their parents annex, often quite unconsciously, many small hints of management and dieting.

### A HANDSOME GIFT.

A valuable and acceptable gift has been formally transferred to the trustees of the Redditch Nursing Association by Mr. S. Allcock. It consists of an excellent Nurses' Home, and the site and cost of erection have been entirely borne by the generous donor. The Association has now been organised for five years, and during that time its value has been highly appreciated in the district, great interest being felt in its progress and prosperity.

### COMFORTABLE QUARTERS.

The Cottage for Sick Nurses erected by the Staffordshire Institution has already proved a valuable adjunct. It contains every accommodation for invalided members of the staff, who are tended by a nurse who has been for some years in the employ of the institution. The advantage of any institution possessing a quiet resting-place for workers who are "a little run down," as well as for occasional cases of more serious illness is too obvious to need comment, and the committee at Stafford are to be congratulated on their cottage.

### SMALL OBJECTIONS.

One of the able-bodied inmates at Ballinrobe Union manages the kitchen, the laundry, and waits on the matron, according to the recently published report of a Board meeting. It seems almost strange that so capable a person should be an inmate of any workhouse, and now that the nurse at the Fever Hospital objects to sleep there alone, she has applied for permission for this useful woman to spend her nights with her in the hospital. The only objections to this were raised by the master, who, if report be true, first said he was not going to inconvenience the house by sparing her from the kitchen, and when a guardian pointed out that she could hardly be needed there at night, he asserted that he was not going to be up at six o'clock in the morning to take charge of her. Surely a woman capable of performing so many responsible duties could be trusted to find her way alone. The guardians finally set the objections aside, and gave the required leave. We wonder if there is any necessity for a nurse to live in the Fever Hospital when there are no patients, or whether some other arrangement is not feasible?

### LIBERAL HELPERS.

The working men at Barry Dock got up an excellent entertainment last week in aid of the funds of the Nursing Association. They not only gave their time to this, but also defrayed the whole of the attendant expenses out of their own pockets. Therefore the proceeds were entirely handed over to the Association, which thus received a handsome donation. Such an example might advantageously be followed in other places.

### NEGLECTED AT NIGHT.

The Lunacy Commissioners have decided that the imbeciles at Dudley Workhouse are inadequately nursed, and it only appears strange that this discovery was not made long ago. Many of these poor people are epileptics, and, according to the local press, there is "no night visiting and no night nurse employed." However, the afflicted are not left quite by themselves during the long and dreary night, for, we learn, "a pauper is placed to sleep in each dormitory." There is a grim humour in the last idea which must strike most readers, and it is a pity that those responsible for such an arrangement have been unconscious of the bitter irony of placing their helpless patients under slumbering supervisors.

### QUEEN'S NURSES IN SCOTLAND.

The Inverness Branch of the Queen Victoria Institute gives a most satisfactory report of the work done by Nurse Nicholson amongst the sick poor. Unfortunately the subscriptions to the nursing fund have not increased at the same rate as the demand for the nurse's services, but the committee look hopefully forward to the results of a well-organised system of collecting during the present year.

### A MEETING AT DROYLSDEN.

A meeting recently held in Droylsden was attended by a large number of ladies and gentlemen anxious to do honour to Nurse Mills on her departure from the Sick Nursing Association. A presentation, consisting of a nurse's satchel and wallet and *Quain's Dictionary of Medicine* was made, and numerous kindly testimonials to Nurse Mills' excellent work were offered by the various speakers. These included Dr. Gellatly, who was in the chair, Dr. Gunn, the Rev. R. Odery, and Mr. Marchington. Nurse Mills' kindness and attention to her patients, and the satisfaction which she had given to the doctors, were specially mentioned, but, being mortal, the nurse has not succeeded in pleasing everybody. The exception, according to the reports given in the local press being the ladies connected with the Association. One of the doctors said "they interfered too much with the work of the nurse." If this is the case the latter is certainly to be condoled with. Kindly supervision is one thing, interference another; and the organisation of a District Nursing Association should include the accurate definitions of power if work is to on without friction.

### THE ALICE FISHER ALUMNÆ.

About fifty members were present at the second annual meeting of this association, the President, Miss Marian Smith, being in the chair. The objects of the association are briefly these: To encourage post graduate study, to raise the standard of nursing, to help those in trouble or sickness, and to promote social intercourse amongst the members. After the formal business had been transacted a pleasant tea party took place.

### HEROIC DEEDS.

A most thrilling account of a nurse's adventure is given in the April number of *The Trained Nurse.* The heroine was on night duty in a cottage hospital, and a tramp, probably tempted by a rumour that there was money in the place, suddenly appeared in the kitchen armed with a knife. An encounter ensued, and the courageous woman actually succeeded in getting the weapon from him and forcing him out through the window by which he had entered. The nurse, however, received some serious wounds, losing much blood, but eventually made a good recovery. The Editor of *The Trained Nurse* invites correspondents to send in accounts of acts of heroism performed by nurses, and we certainly think that nothing could be more interesting than such narratives. We shall always be glad to give prominence in our columns to any well-authenticated deeds of heroism achieved by nurses.

### HELPING OTHER NURSES.

Every bright spring day tends to make nurses think of their well-earned holidays. Whilst some have homes where their rare visits are eagerly anticipated, others less fortunate have to look out for a resting and refreshing scene in which to recruit for another year's work. From time to time we get a great deal of information regarding the places favoured by our readers, and the general surroundings, excursions, &c. Yet descriptions of her own past holiday are of little practical value to a nurse's companion unless she enters into details. For instance, it is possible to be of real use to many nurses in all parts of the country, a very little personal trouble enabling one traveller to furnish others with all details as to cost of a past tour and its difficulties, not only the charge for railway tickets, which is easily learnt, but the rate charged for hiring vehicles, boats, &c., and the approximate cost of living and of lodging in pretty country, seaside, or mountainous places. During the months when there are fewest visitors things are cheapest everywhere. In a word, if a concise statement of the whole cost of a holiday (to a person of simple tastes and moderate means) were sent in the form of a letter to The Hospital, it would prove acceptable to many.

### A CONVALESCENT HOUSE.

The convalescent house belonging to the Edinburgh Royal Infirmary, bearing the name of "Corstorphine," was first opened five-and-twenty years ago. Now that new wings have been added to the building it is estimated that about 1,400 patients will annually be received for a three weeks' visit, and this period can be extended if desirable. The Royal Infirmary patients always have preference, but when there is sufficient room other persons requiring change of air are admitted if considered suitable cases by the medical officers. The convalescent house stands in a park of six acres, and is beautifully situated.

### SHORT ITEMS.

A successful concert at Trentham resulted in a satisfactory addition to the Nurses' Fund.—At the ball given at Cardiff for the Nurses' Home, the refreshments, which were unusually good, were provided by the Ladies' Committee.

### On General Nursing.

By Rowland Humphreys, M.R.C.S., L.R.C.P.Lond.

### VIII.—PNEUMONIA.

This term merely means inflammation of the lungs. There are several forms of inflammation of the lungs. Thus there is the form called acute pneumonia, another form called broncho-pneumonia, a third known as chronic or fibroid pneumonia, a fourth known as hypostatic pneumonia, and then there are varieties of each of them. Usually by pneumonia is meant acute pneumonia, the other forms being given their proper appellations.

It is proposed to confine the principal part of this article to the description of the acute disease, and to give a shorter account of the principal varieties of the other inflammatory diseases of the lungs.

Acute pneumonia is a disease which may be very aptly compared to erysipelas. It commences suddenly at one particular part of the lung, and spreads thence to other parts of the same or of the opposite lung with all the symptoms of a severe blood poisoning, combined, of course, with the special ones due to the locality affected, and after running a pretty definite course, it disappears as quickly, in many cases, as it came. To describe it at length, it is divided into stages which depend on the changes found in the lungs in persons who have succumbed to the disease at the stages in question. They are:—

1. The stage of hyperæmia, in which the lung is overcharged with blood at the place where the inflammation is taking place, and exudation into the lung tissues and alveoli.

2. The stage of red hepatisation, where the exudation of red and white blood-corpuscles and of the serum of the blood having taken into the alveoli (the air space) and into the smaller bronchial tubes near the alveoli, the exudation is rapidly becoming solid from the formation of fibrin, just as blood when shed coagulates.

3. The stage of grey hepatisation, which is very similar to the last, but the red colouring matter of the corpuscles having been absorbed, the appearance alters to a greyish tint. At the same time the vessels in the walls of the alveoli which have been distended with blood now being pressed on by the coagulated exudation are almost emptied, and the colour due to the blood circulating in them is, of course, lost. The lung becomes firmer, and a large amount of white corpuscles make their appearance. These come from the blood, and from subdivision of those already shed, and from the walls of the bronchi and alveoli. The fibrin disappears from the coagulated material.

4. The third stage may pass on to that of purulent infiltration; that is, the lung becomes partly or, as a whole, soaked with pus.

5. The stage of resolution or, rather, of reabsorption and expectoration of the coagulated material which has filled the alveoli and smaller bronchial tubes.

We will now pass on to consider the clinical signs which characterise these stages, and at the same time the description already given of them will be further enlarged. Of course, what the physician has to do in these as in all other cases is to recognise the meaning of the signs and symptoms he observes and the pathological changes they indicate. Then the dangers which may be expected are almost a matter of common sense; at any rate, to anyone who knows the elements of disease and of physiology.

The stage of hyperæmia is a very early one in the disease, but it is preceded by that due to the invasion of the complaint. The first symptom is very often a rigor or an attack of convulsions, or of vomiting. These symptoms are of course common to all forms of blood poisoning. The next thing noticed is a rapid rise of temperature. Usually within twelve hours the temperature has risen to between 103 to 105 deg. Fahr., and the respiratory and pulse rates have risen in proportion. There is often pain in the back, or side, or head.

In about one-fourth of the cases the onset is preceded by minor symptoms, such as shivering, or a general feeling of not being well, but the first marked symptoms are those already enumerated. In some cases the onset is gradual, not acute, and the disease then has more the appearance of typhoid fever; indeed, it is often the first symptom of it. Soon the symptoms due to the local disease appear. These arise from the inflammation of the lung and of pleura, the membrane which covers it and whose smooth moist service allows it to move freely in respiration.

Pleuro-pneumonia is the term employed to describe a marked degree of pleuritic effusion combined with inflammation of the lung.

Pleurisy is said to be always present, sooner or later, in pneumonia; it will therefore be described somewhat at length under the head of complications. There is no doubt but that it is the principal cause of the pain in pneumonia, though there are others which will be referred to later on.

The symptoms due to the inflammation of the lung manifest themselves somewhat in the following order. The first sign is often some amount of uneven jerky respiration heard over a limited area in the chest. This area is that which later on becomes solid. Then the cough comes on. It is short, restrained, frequent, and hacking. At first dry, it soon becomes moist, and a clear, jelly-like mucous expectoration makes its appearance. The respiration-pulse ratio is altered from the normal. In health and in simple fevers the respiration is to pulse as 1 to 4 or 4½; in pneumonia it soon becomes 1-3 or 1-1½; in other words, the respiration rises to between 40 and 50, while the pulse only rises to 120 or so, instead of to over 200, as it would do if the normal ratio were kept.

The *alae nasi*, the nostrils, expand markedly with each inspiration. The face is flushed, perhaps slightly jaundiced, and the angles of the mouth are often blue, the skin burning hot and dry. Speech and swallowing become difficult from the rapid respiration. Mental symptoms develop, the patient complains of headache, and becomes very restless or delirious. Sleeplessness is usually markedly present, and is one of the most serious troubles to deal with in the early stage of this disease. The urine is scanty and high coloured, and usually contains albumen, and there is a total absence of sodic chloride, or common salt, which is normally present in considerable amount.

Usually about the second day the physical signs of effusion into the alveoli, lung substance, and bronchi make their appearance. The ear hears sharp, fine crepitation; a sound which has been described as like that produced by rubbing hair between the fingers; it is also very like that made when air is sucked in between the moistened, closely-shut lips, especially if the ears are closed at the same time. It is due so the swollen condition of the lining membrane of the smaller bronchi and of the alveoli, the air bubbling through the moisture already exuded into them.

This fine explosive crepitation at the extreme end of respiration is characteristic of this complaint, and although it is found under other circumstances, such as in pleurisy and in capillary bronchitis, yet it is persistent only in the disease we are speaking of, and it is soon followed by the signs which indicate consolidation of the lung.

### Wants and Workers.

*Nurse B.* will be glad to know if there is any place in the central part of London where nurses are lodged and boarded at moderate terms during quarantine after scarlet fever.

### The Friends and Foes of Nurses.

THE ROYAL NATIONAL PENSION FUND FOR NURSES v. THE RECORD PRESS (LIMITED).

Much misconception having arisen from the statement in some newspapers that the trial of this action resulted in a "compromise," it may be well to state what actually occurred. The action was for an injunction to restrain the further publication of the libels and for damages.

Mr. Finlay, Q.C., the leading counsel for the plaintiffs, having said in his opening that the plaintiffs wanted the "case tried in such a manner that nurses should know the nature of the attempt made to deceive them," Mr. Justice Bruce at a later stage said: "What you really want, Mr. Finlay, I take it, is to preserve the credit of the institution. If you get the character of your institution established the actual damages would become immaterial. All you want is to have the character of your institution established." To which Mr. Finlay replied: "With this institution damages are nothing. All they want is to let the nurses be warned against these attempts which have been made to mislead them." Mr. Justice Bruce: "Could you not arrange now without the expense of a trial? The defendants, I understand, say they have no defence. The question as to the actual amount of damages you may be entitled to recover is probably somewhat difficult, but your object is not to recover a large pecuniary sum, but to establish the character of your institution." Mr. Finlay: "Certainly. I make again the offer which we have made already, and which the defendants have refused. If the defendants will make a proper apology, to be sufficiently published, and pay the costs of these proceedings, the proceedings will be terminated." Mr. Justice Wright: "We have power, I think, to attach any terms to the leave to withdraw the defence. Any condition that the Judge ought to impose can be imposed here, I suppose, or by the Court of Appeal."

The terms of the Court included:—

1. An injunction restraining the further publication of the libels.
2. Damages 40s., and
3. An apology and costs of the action.

The form of the apology has been settled by Mr. Justice Wright, and runs as follows:—

"In the High Court of Justice.—Queen's Bench Division.

"The Royal National Pension Fund for Nurses v. The Record Press (Limited).

"Public Apology.

"We, The Record Press (Limited), the proprietors of the *Nursing Directory* and the *Nursing Record*, hereby express much regret that we have published statements to the effect that the Royal National Pension Fund for Nurses had been and was in the habit of charging nurses from 20 to 26 per cent. higher premiums than nurses would have to pay for similar advantages at old-established London offices.

We hereby acknowledge that those statements are without foundation or justification.

"We have satisfied ourselves that nurses insured in the Royal National Pension Fund for Nurses are able to secure substantial and exceptional advantages.

"We have consented, and do hereby consent, to the application of the Royal National Pension Fund for Nurses for an injunction restraining us from repeating those statements in any publication belonging to us or under our control, and also to judgment for the sum of 40s. damages, with costs.

"Dated this day of April, 1894.

"The Office of The Record Press, Limited.,

"376, Strand, W.C."

With reference to the publication of the apology, Mr. Justice Wright said at the hearing:—"It is to be distinctly understood that the plaintiffs are not to run any risk by advertising the apology wherever they like and as much as they like." Mr. Lynden Bell (counsel for the defendants): "They must only state what is the fact." Mr. Justice Wright: "Whatever your apology is they must be at liberty to use it as much as they please." Mr. Lynden Bell: "Certainly, my lord."

### The Indian Nursing Service.

(By Our Own Correspondent.)

The correspondence that went on for some months in *Truth* on the propriety of nursing sisters taking part in dances must have attracted at least a few of your readers' attention. We cannot but regret that one of our number was so unladylike as to publish a "confidential letter" without permission. The Commander-in-Chief did not express his wishes through Surgeon-General Madohan without sufficient reason, and doubtless he felt as others did that some little restriction should be laid on the amusements of the nursing sisters. Last year a number of nursing sisters were on the sick list, not so much from overwork as from overdoing it with work and gaiety combined.

With all the so-called arbitrary restrictions laid on the sisters, they have much freer and more enjoyable times than any nurses in England, and for three months in the spring during the healthy time they have a life of rest and leisure. Nurses who are "too delicate" to do night duty do not consider themselves too delicate for night amusements. There are many nurses willing to give or be given in noble work. Miss Loch, lady superintendent, has returned from one year's furlough in England to Rawal Pindi; Miss Betty to Meerut; and Miss Maxwell Muller, on promotion to Bangalore, as lady superintendent Madras circle; Miss Harris, department superintendent, returns to Peshawar; Department Superintendent J. M. James goes from Rawal Pindi to Rangoon, Department Superintendent Murray from Bangalore to Mian Mir, Department Superintendent D. M. Moore from Meerut to Quetta, and Sister L. Jardine from Rangoon to Lucknow; Nursing Sister Cann from Mian Mir exchanges with Nursing Sister Hayes from Peshawar.

### The History of Nursing.

Amongst many interesting articles in our American contemporary, *The Trained Nurse*, there appears one by Dr. Henry Sewall on "Nursing, Past and Present." It was originally addressed to the graduating class of the Colorado Training School, and it gives many interesting details of the tendance of the sick in early times. Probably some limitation of time prevented Dr. Sewall from giving at the moment a more exhaustive account of the subject, but readers cannot fail to regret the absence from this paper of accurate data regarding the progress of skilled nursing, the growth of the art in the writer's own country, as well as in our own, being most sparingly touched upon. Dr. Sewall speaks of only attempting "to give in outline a sketch of the past history of nursing," but we sincerely regret that he has not succeeded in giving a sufficiently concise and definite summary of the whole of this subject in his pleasantly written article.

### Women Volunteers.

The accompanying sketch represents the uniform which it is proposed to adopt in the newly-formed Women's Volunteer Medical Staff Corps, and has been made from a description kindly given by Miss Ethel Stokes, hon. secretary to the corps. It will be seen that the obnoxious petticoat, the bugbear of advanced womanhood, has been altogether dispensed with, the costume to consist of a tunic and full knickerbockers made of tweed or some other serviceable material, in which blue will be the prevailing colour. Box pleats down both front and back will give a slight fulness to the skirts of the jacket, while the ordinary stand-up collar of the soldier will be eschewed as both inconvenient and unhealthy, its place being taken by the "turn-down" species; other variations on the customary military equipment being the shoes and gaiters. The chief difficulty at present is with regard to the hat, authorities being somewhat at variance as to the ideal military head-gear. The straw hat is one suggestion; to be of the ordinary "boating" shape, with the badge of the corps worked upon the ribbon, in much the same way as the arms or crest on a college cap. The forage cap is also under consideration; while one military man to whom the matter was mentioned gave it as his opinion that if the ladies wanted a brim they should have felt hats, after the manner of the picturesque head-gear adopted by many of the Italian regiments and by the New South Wales Lancers, whose "get-up" attracted so much attention at the opening of the Imperial Institute. This would be at once practical and picturesque, would give a distinctive air to the corps, and would be more accommodating to circumstances than the unbending straw.

Women Volunteers—Proposed Uniform for Officers and Privates.

At the meeting held at the Ideal Club more than one (male) speaker declared himself certain that, whatever costume the fair volunteers decided upon, they would be sure to look charming; while the originators of the movement themselves make no secret of the fact that they hope by their example to advance the cause of rational dress. Probably, if they fulfil the prophecy of their male critics, they will succeed in this endeavour; they are at least plucky in attempting it. The first lady who summons up courage to walk down Oxford Street with her sword dangling at her gaitered heels will deserve our admiration, perhaps, but also our compassion—unless, indeed, she happen to be short-sighted and to have left her spectacles at home. It may be some comfort to intending recruits to know that they will not be expected to come up to the military standard as regards hair, or rather the lack of it; but we are not aware whether any fixed and uniform method of hair-arrangement will be required from members of the corps. Doubtless this and many other little details will be settled when the preliminary arrangements have been satisfactorily made, and the corps is in full swing.

After all the Salvation Army has paved the way for female warriors, and we shall in a very short time get used to captains innocent of moustache, majors who wear their hair in a bun at the back, and colonels in—no, not petticoats—rational dress! It is merely a matter of overcoming prejudice. If the lady volunteer has no objection to affording material for jokes to the journalist, and for immense delight to the boy in the street, there is no reason that she should not become a recognised fact; and as one of the characteristics of the advanced woman is her stoical indifference to ridicule of any kind, it is very probable that a fact she will become.

It is hardly to be expected that the military authorities, at any rate at the outset, will recognize the newly established force, which will have to struggle along without the assistance of the customary capitation grant; while probably a good many difficulties will be thrown in the way of attending manœuvres, &c., as the corps will probably wish to do, and indeed should do if it is to be of any use in real warfare. Whether the strength of women is equal to the work is a matter which experience alone will decide; but the organizers of the movement will do well to insist upon a somewhat high physical standard for intending recruits. Women who intend to transport their fellow-creatures on stretchers should be of a strength, if not a height, above the average; or there will soon be complaints about the inefficiency of the corps.

### The Mutual Relations of Nurses and Medical Women.

By Mrs. Scharlieb, M.D., B.S.

### II.—TEACHER AND PUPIL.

3 and 4. These divisions of the subject may be considered together, for if the attitude of mutual repulsion attributed to the fully-trained nurse and the awkward, self-conscious girl be unfortunately a fact, it is only natural that they should not voluntarily assume one of the sweetest relationships of life—that of teacher and pupil. I am afraid that in many instances there is a want of friendliness and cordiality on both sides. Unfortunately, even devotion to the sacred cause of medicine, whether as doctor or nurse, does not kill within our hearts that energetic descendant of the old serpent, known as "self-love." If we will be honest with ourselves we must acknowledge that this terrible love of self (leading to ambition, detraction, and pride in some of us, and to jealousy, unreasonable aversions, and censoriousness in others) is at the root of the difficulty experienced in working harmoniously, especially where the workers are divided into classes. First, to take the trained nurse, who is said by the writer to "invariably cherish an instinctive aversion to the awkward and self-conscious young girl who is clumsy over her dressings and has not the most elementary idea of putting the patients' comfort in the first and her own studies in the second place." Upon the writer's own showing the nurse brings to her work an additional experience and womanliness of some half a dozen years. She begins as probationer at 23, and is probably not staff nurse until three or four years later; so that, compared with the average medical student, she is a woman of position and experience, who should be both able and willing to direct and assist. What we do constantly we do well, or should do well, if we did not stereotype our imperfections; and the nurse whose natural intelligence and general education is frequently as good as any student's, should have so profited by her special training that in her own branch she should be technically perfect. This in many instances she is, and she needs not to learn her art, but that most excellent gift of love. Being so fully mistress of the situation, she can afford to be generous, and to help those who are now what she and all of us have been—"clumsy and self-conscious" neophytes. If nurses could and would act in this way they would generally secure the welfare of their patients and the gratitude of the students.

The women medical students are unfortunate frequently in proportion to their cleverness—and their youth. The bright, hard-working girl, who has distinguished herself at school and college, and who has found the facts of anatomy and physiology no more formidable than were mathematics and Greek verbs, comes to hospital knowing nothing of the "sweet uses of adversity," and, like the rest of us, she is none too willing to undergo discipline. She finds herself in a new sphere, in a world where anatomy and physiology are modified almost beyond recognition, and where the drugs seem to exhibit alien properties so little do their results accord with the predications of materia medica. If she be a dresser she brings to her duties an absolute ignorance which is most paralysing, and which no one seems to be able and willing to enlighten. The house surgeon gives her a few rapid directions, and leaves her to carry them out. Her seniors are busy, and occupied with their own duties; the nurse is at least not likely to offer help. What wonder that she is awkward and does badly. Girls are more self-conscious than boys, and are prone to think that everyone's eyes are on them, that their failure or success matters much to others. We have all passed through this stage (except those who never get beyond it).

All this is readily understood by sensible people; the foolish and wrong thing is when the neophyte, instead of frankly acknowledging her ignorance and asking for help, stands on what she conceives to be her dignity and "looks down," as we are told, on the nurses. Does it not occur to the writer that women students like women nurses are drawn from several ranks of life, and from families of varying traditions? and that whereas a girl brought up in a well-ordered home, trained to be courteous to all, will at once give honour to whom honour is due, another girl, as clever and as able to distinguish herself in examinations, may be destitute of inherited or acquired politeness. No doubt the inevitably hard work is made harder to these latter students, although they need help and training more than do the others. Now that the medical profession is open to women there will be a period during which some unsuitable students will enter, just as there is a certain proportion of male medical students who have entirely mistaken their vocation. Of these unsuitable students some fall out of the ranks during training, others qualify but never practice, and a few are trained into useful practitioners, although at the cost of much suffering to themselves and to all concerned in the process.

The blame, then, of the "strained relations" must be divided, and both nurses and students should earnestly strive after the "more excellent way" after a cordial co-operation, which will advance their own interests, the welfare of their patients, and the glory of God.

5. "That a woman should be before the other sex in courteous demeanour; but alas! she is not so."

This is a very serious charge, and one that admits of no refutation except that of time. The qualified medical women number as yet only 200 or so, but every year adds to their ranks, and it rests with themselves, and with themselves only, to prove that education has not been to them a curse rather than a blessing. It is to be remembered that woman's education is passing through a transition period, and that all periods of change are seasons of peril; that old things are passing away, and that new things are not yet secured. For some years past so much fuss has been made over a girl who could do the same as her brothers that the young woman has been almost justified in believing what everyone told her—that she was a very remarkable person. Now things are slowly settling down, and we no longer feel astonished and proud that our girls should be normal human beings. The result will be good for our girls, who will soon find that to be well educated is no more remarkable than to have the usual complement of fingers and toes. Then when we are less lost in wonder at our own attainments we may hope to regain the old womanly sweetness and courtesy that distinguished the gentlewoman of bygone days. Our brethren of the profession are heirs to a good name for generosity, and for that consideration for others that marks a great physician. Shall we women be content to be behind them in this respect? Why should we not rather, both nurses and doctors, join in a noble strife to live up to our highest ideals, and to hasten the day when the best possible shall be done to avert disease and to heal the sick?

### Appointments.

The Hospital for Sick Children, Great Ormond Street.—Miss Florence Smedley has been appointed Matron of this hospital. She was trained at St. Bartholomew's Hospital, held the post of Ward Sister there for five years, and was afterwards Matron of Swanley Convalescent Home. We wish Miss Smedley success in her new field of work.

Swansea Fever Hospital.—Miss Jeannie Land has been appointed Matron of this hospital. She was trained at St. Pancras Infirmary, London, where she remained for nearly four years, then did district nursing in Leeds, was Night Sister of enteric wards, Sister-in-Charge of scarlet fever wards, and Sister of small-pox wards at Leeds City Hospital. We sincerely congratulate Miss Land on her well-earned promotion. Her testimonials are excellent.

### Nursing in Philadelphia.

(From a Philadelphia Correspondent)

There has been (as Daudet puts it) a plentiful lack of news lately, but the last few weeks have made up for the quiet of the winter. To begin with our own school, we have at last succeeded in abolishing the one-year course, which was only started to eke out our supply of nurses in the hospital, the money appropriated for "wages" not being sufficient to secure enough nurses to do the work properly. So these one-year girls were paid nothing, though they were boarded and lodged free as the others. Now all come for two years, which will be a great improvement in every way. I am sure it is the same in England as here, that with medicine and its progress, the demands upon nurses increase accordingly, so much so, that the hospital of the University of Penna has just prolonged its course of nursing to three years instead of two. I said in my annual report of the training school this year, that "it is not too much to say that the details of scientific nursing have doubled within the last ten years." We have a flourishing Alumnæ Association a year old, with two hundred members. It bears the name of the honoured founder of the school, to whom we all owe our training and diplomas and the standard our school bears to-day—the Alice Fisher Alumnæ of the Philadelphia Hospital Training School. On Easter Monday the second annual meeting took place, and we adopted our constitution and bye-laws. I was greatly helped in the making up of these by the volume on "Hospitals, Dispensaries, and Nursing," the report of the International Congress of Charities and Correction at Chicago, June, 1893, which has many valuable papers in it. We hope in time to have a house in town which shall bear the alumnæ's name, a sort of club, and more than that, a home—lecture rooms, &c., downstairs and bed-rooms upstairs—with a graduate nurse in charge of the whole. One of our bye-laws is to the effect that we wish to encourage "post graduate study." We have 500 graduates, and I feel we can and shall be a power if we work in the proper spirit, and all pull together. We had to rather alter our Easter Day programme this year on account of the rain. Each year we have sung the same three hymns, two of which were sung at Miss Fisher's funeral, and the other her favourite, "The King of Love, My Shepherd Is." It always surprises me when each year the nurses are asked if they will contribute a small sum towards flowers for her grave, that in a few hours from twenty to thirty dollars are collected, all given by women who never saw her face! So much for the lasting memory of a noble woman. Surely nothing that bears her name can be a failure! I fear the news and affairs of our own school will crowd the others out, so I will stop here, and go on about the other hospitals. The University Hospital had a commencement in February, and addresses were made by the Rev. Charles Wood, Presbyterian minister, Dr. Horatio C. Wood, professor of therapeutics, and Dr. Barton Cooke Hirst, professor of obstetrics, who made the address of the evening. The advice given to private nurses on the subject of discretion in speech and the value of "holding the tongue" was excellent, practical, and very much more to the point than the average address consisting of flowery language about smoothing sick pillows and good wishes. The diplomas were tied up with the 'Varsity colours, red and blue, and the amphitheatre prettily decorated with flowers—between the speeches an orchestra played. Ice cream was served later to the guests, and an invitation to inspect the pretty Nurses' Home was accepted by most of the people present. The rooms of almost all the nurses were beautifully filled with flowers, chiefly roses, and the intelligent faced girls and brightly-lighted house made a charming picture, and to those who only saw that side of a hospital nurse's life it must have appeared a very much more merry one than to us who know something of the struggle for patience and for success, and the long dark nights of watching, and the trials and temptations to overcome the weariness and disinclination to study, which come more or less to all of us. The Penna Hospital had its graduation in March, eight nurses receiving diplomas; Dr. John H. Packard, surgeon to the hospital, gave the address. After the exercises the graduates and their friends adjourned to the Nurses' Home, where refreshments were served and a dance given. I hope this letter is not too long, and that something of interest will be gleaned by readers of The Hospital. Our Congress of Superintendents of Training Schools, held in New York in January, was a great success and most enjoyable.

### Notes from Melbourne.

(By Our Own Correspondent.)

The "Massilia" arrived here on March 3rd with two cases of small-pox on board, and the passengers were at once put in quarantine. No further cases have developed. The period of detention is regulated by the vaccination or non-vaccination of the passengers as soon as the cases were discovered on the "Massilia." Melbourne, Sydney, and Adelaide are the only Australian ports possessing quarantine stations, and it has been suggested as highly desirable that such a station should be established at Cooktown (north), and Albany (west) respectively. A case of small-pox has just been discovered at Perth (Western Australia) in the person of a half-caste girl. There are said to be no evidences of contagion, and the curious are speculating upon the probability of the disease having sprung up *de novo*. When Australia was discovered some of the tribes were found disfigured by traces of small-pox, or some disease that pitted the skin in the same way.

Victoria has taken a step forward in the right direction, having resolved to appoint women factory inspectors. But in accordance with the policy of the colony these women become civil servants, and thus we find the Public Service Board inviting applications from women in the service in the non-clerical division to fill these positions. Applicants must be not less than 28 and not over 40 years of age. The salary is fixed at £130, increasing to £150 per annum. The clerical division includes those employed as postmistresses, telegraph operators, or clerks and assistants in post offices. The non-clerical division includes matrons and warders in lunatic asylums and gaols, as well as the great body of women teachers in the State Schools of the colony. Doubtless some amongst them will be found to have special knowledge of sanitation, but few can have had any training therein. A trained nurse is in charge of the Melbourne gaol hospital, and another is at the head of the sick ward on the female side of the Metropolitan Lunatic Asylum, but there are few, or none, in the country gaols or asylums.

The Melbourne District Nursing Society (for nursing the sick poor in their own homes), the only association of the kind in the colony of Victoria, has just completed its ninth year of existence, and announces its annual meeting. From an advance copy of the report we find that, though suffering from diminished revenue, like all other societies at present, the Society has been extending its sphere of usefulness. For the first time three trained nurses have been employed continuously throughout the year, and have paid 14,382 visits to 703 patients. Further, it has been resolved to undertake midwifery cases among the very poor who may not be able to avail themselves of the Women's Hospital or procure medical skill. For the services of the head nurse (duly trained in the Women's Hospital), who undertakes this branch, it is proposed to charge a small fee, by which means it is hoped ultimately to make this branch of the Society's work self-supporting. It should be remarked here that patients are charged one shilling as registration fee and subsequently one shilling per month. These fees are not enforced in every case, however. The Society has taken another new departure in the establishment of a course of popular lectures on Hygiene, to be given by medical men. The first series of the course have already been delivered during February by Dr. W. Atkinson Wood. These lectures, on "The Prevention of Sickness and the Treatment of Children," were given at the Home of the Society in Carlton, and were attended by a large number of mothers, and Dr. Wood, for further instruction, presented the Society with 500 printed instructive cards for distribution. The affairs of the Society are administered by a committee of ladies and gentlemen. It receives no subsidy from Government, but participates in the Hospital Sunday collections, and does not employ a collector. The nurses are not supposed to attend infectious disorders, but as a matter of fact three cases of measles and one of scarlet fever figure on their list, which is headed by 100 cases of rheumatism. Why rheumatism should be as rife in the dry climate of Australia as it is in damper countries has long been a matter of curious speculation.

The rate of typhoid continues high, the number of cases for the whole colony in a recent week being 138, with 15 deaths.

### A Spanish Hospital.

Some ten years ago the Provincial Hospital of Huelva was in a sad condition of insanitation, the theory of antiseptic surgery being so little understood that too frequently so-called antiseptic dressings were put on with dirty hands, and in the same wards were treated cases of typhoid, malaria, tuberculosis, &c. No precautions were taken to disinfect the sputum of the phthisical, and bed-clothes which had been used for a patient with some infectious disease, served after only an ordinary washing for a surgical case with open wounds. Protests were made by the medical men, and the Provincial Deputation in consequence caused plans for a new hospital to be drawn up, but here the matter has so far ended.

Now this was the only place to which the injured and sick amongst the workmen of the Rio Tinto Company could be sent, and many lost their lives from preventible diseases, septicæmia and erysipelas. Dr. W. A. Mackay, the medical man attached to the company, found insuperable difficulties in the treatment of cases, having to contend either with the dirt of the men's own houses or the even worse conditions of the hospital. He therefore represented the matter strongly to the directors of the company, and the result was an order for the erection of a hospital for the use of all workmen employed by the company, the sailors from their ships, and convalescents from Rio Tinto.

Dr. Mackay himself directed the plans, and supreme care was given to every minute detail, so that every possible hygienic advantage might be obtained. An excellent site was obtained on the outskirts of Huelva, high and isolated. A large garden is attached, where the convalescent patients spend most of their days. The building is light and airy, and conditions of almost perfect cleanliness reign. It is so attractive in appearance that the beggars who pass by its gates are hardly to be convinced that it is actually a hospital, such a building in their experience being associated with very different ideas, saturated with evil smells and looking like a prison.

The building consists of two large wards, rectangular in shape, 44 feet in length by 22 feet in breadth, attached by corridors to the centre administrative block. Each ward contains ten beds, and it is calculated that 1,400 cubic feet of air may be allowed to each. The wards are each lighted by seven windows, and a perfect system of ventilation prevails throughout. The floors are of asphalt, impermeable to germs. Two small isolation wards open from the corridor. In the administrative block are the consulting and operation rooms, bath-room, and kitchen. A sun-room is made of a verandah, shut in with glass, and opening into the garden. The upper part of the hospital extends only over the administrative portion, and contains rooms set apart for English patients, sitting and bed rooms for the nurses, servants' rooms, and stores.

The management of the house and the superintendence of the nurses and servants is under the charge of an English lady, Miss Blackadder, a certificated trained nurse, who with Miss Ferrier, another English nurse, has won golden opinions from doctors and patients. The following expression of opinion comes from a Spanish doctor connected with the hospital:—

"This is a career (that of trained nurse) which does not exist in Spain, and which requires three years of study, theoretical and practical, in a hospital." . . I have the highest opinion of these nurses. These women unite the heroism of sisters of mercy with a technical education which gives them an intelligent interest in their work, and in the brother who suffers a severe surgical operation they see not only the life of the patient at stake, but a scientific problem to be solved, and the advance of science to be furthered. . . All honour to such work. I would that we might see a similar career established in Spain."

Two Spanish nurses form part of the staff, and there is a sufficient number of servants and helpers to keep the hospital in good working order. The kitchen department receives much attention, "good meat and wine, pure air, and sunlight" being rightly held by Dr. Garcia, who has charge of the management, to be the most powerful tonics procurable.

A good work is evidently being done by this little hospita and its energetic staff. It is only to be hoped so excellent an example may have the effect in due course in stirring up the Spanish authorities to some conception of the proper treatment of the sick.

### Minor Appointments.

Brentwood Cottage Hospital.—Miss Baker writes to say that she is merely taking temporary charge of this hospital, and that she was for three months acting as Matron at the Dorset County Hospital.

Devon and Exeter Hospital.—Miss Jessie Sargent has been made Housekeeper at this hospital, having previously held the post of Matron at Ryde Convalescent Home, Isle of Wight. Miss Sargent takes many good wishes with her to her new work.

Up-Country Nursing Association for Europeans in India.—Miss Jane Blanche Becks has been appointed Nurse to this association. She was trained at Beckenham Cottage Hospital, Tewkesbury General Hospital, and St. George's-in-the-East Infirmary. Miss Becks will take with her many hearty good wishes.

### Presentations.

On his resignation of the post of Surgeon at the Carlow County Infirmary, Mr. O'Callaghan was entertained at a farewell supper by the members of the Carlow Masonic Lodges. An illuminated address was presented from Lodges 116 and 91, and some grateful patients gave a most beautiful clock with Westminster chimes. Many kind speeches were made and universal regret expressed at Mr. O'Callaghan's departure for London.

Miss E. Burrow on leaving Bolton Infirmary, where she has worked for the last five years, was presented with a charming afternoon tea service, a pair of flower vases, and other gifts from the resident and nursing staff.

The lady superintendent and nurses of the Wakefield Home for Hospital Nurses presented Mrs. Waddy with a handsome silver fruit dish, on her resignation of the post of matron.

☞ For Everybody's Opinion, Reading to the Sick, Book World for Women and Nurses, &c., see page xxix. et seq.

### The Muses' Looking Glass.

### WHERE TO SPEND A HOLIDAY.—LANERCOST PRIORY.

After leaving Naworth Castle the holiday-seeker will find a delightful way through the woods leading to Lanercost Priory.

Like nearly all old monastic buildings, it lies in a valley, for it is curious that our forefathers almost always loved to build in a sheltered valley, while we think a hill, or at all events a hill-side, the best possible place for a house. Our ancestors could not run off to Cannes or Mentone to escape from the east winds, to which we in our breezy dwellings are exposed, consequently they chose quarters that were good to live in all the year round, and their pleached alleys and paths shaded by huge yew tree hedges provided them with sheltered walks.

The nave of the Priory is roofed in, and serves as an unusually grand parish church, with fine Norman pillars and clerestory. In the church is preserved all that remains of the churchyard cross, a finely-carved fragment about three feet high, with a good bit of dog tooth running up its four edges. The inscription tells that it was put up by the monks to celebrate the removal of the interdict under which England had languished for five years in King John's reign; but over the monkish Latin a more modern legend tells that one Sarah Smith, being of an economical disposition, used the broken fragment as a tombstone for herself, and her virtues are written across the original inscription. The tourist is grateful to her, however, for others would have appropriated the stone if she had not, and it might now be serving as a gate-post or a drinking-trough.

Through the east-end of the church the transepts and choir of the Priory are entered; the walls and windows are intact, but the roof disappeared long since. Sir William of Triermaine, familiar through Sir Walter Scott's "Bridal," is buried here, and many of the old Dacres and Howards lie in grand square tombs, which have long since lost their recumbent figures. Some of these tombs were broken into between eighty and ninety years ago, and the lead coffins stolen, to make, it was presumed, bullets for use against Napoleon when all England was arming in terror at his threatened invasion.

Extensive monastic buildings still remain, and serve as stabling, &c., for the rectory, while the old refectory, with its fine Norman crypt, still partly fulfils its original purpose, being used for parish teas and entertainments.

The rectory itself teems with historical associations, and is with the Priory the goal of many an American pilgrimage, for it was there that Edward I. came on his way to conquer Scotland, and where he again stayed with his Queen when seized with his last fatal illness, Burgh-on-Sands, where he died, being not far off.

### Notes and Queries.

### Queries.

(43) *Midwifery.*—Where can I get free training in midwifery?—*Alpha.*

(44) *Incurables.*—I shall be glad of information about Homes for Incurables, and how admittance is gained.—*Sister Helen.*

(45) *Water.*—What is the best way to keep water sweet in fire buckets, and obviate frequent changing when water is scarce?—*S. S.*

(46) *Male Nurse.*—Where are male nurses trained, and what fees do the hospitals require?—*A. E. D.*

(47) *Training.*—Information about Liverpool hospitals wanted.—*Sweetbriar.*

### Answers.

(43) *Midwifery (Alpha).*—We do not know of any place where you can get free training in midwifery if you have had no general training.

(44) *Incurables (Sister Helen).*—See Burdett's "Hospital Annual," published by the Scientific Press, 428, Strand.

(45) *Water (S. S.).*—Probably a little charcoal will answer your purpose.

(46) *Male Nurse (A. E. D.)*—No general hospitals in England take male probationers.

(47) *Training (Sweetbriar).*—See answer to 44.

### Notes from America.

Seven nurses graduated this spring at the Newark, N.J., City Hospital, and all took high honours. This is a lower number than usual, but several probationers were unfortunately obliged by ill-health to resign before the completion of their two years' training. The honour of inaugurating the first American training school in connection with a religious community belongs to St. Mary's Hospital, Brooklyn, which did so in 1889, and the example has been followed in the Carney Hospital, Boston, in Buffalo, and in St. Vincent's Hospital, N.Y., all the schools being under qualified superintendents. Training schools are multiplying daily over here, and one of the latest is in connection with the Hahneman Hospital, Philadelphia. A new nurses' home, one of the finest in the country, has been opened this year at Woodland, Mass., in connection with the Newton Hospital. It is an exceptionally fine building, admirably situated. Nurses here seem somewhat tired of the subject of the National Badge, and indeed it has been discussed to a rather wearisome extent, although no decision appears to be as yet arrived at concerning it.

### Where to Go.

### Mrs. Allingham's Water Colour Drawings. The Fine Art Society's Galleries.

Mrs. Allingham's water colour drawings, whilst treating Nature in a charming manner, treat her undeniably on her smaller side; indeed, amongst this collection one looks in vain for breadth of subject or breadth of treatment. The rural homes of England, quiet dells and nooks bathed in summer sunshine, are subjects which exclusively monopolise this artist's attention. In the isolated examples where she deviates from her regular scheme, success by no means crowns her efforts. These water colours are supremely insular in their virtues and in their faults. They are very sweet, very lady-like; pictures which undeniably are the outcome of a a refined and happy mind, free, too, of the taint of morbidness which stamps itself on the art of 1894. But the sense of their limitation forces itself upon one. Baby faces leaning over motherly shoulders are charming in their way, but in a small exhibition like this one, eighteen examples of this theme are a little over sufficient. That is to say, out of seventy-four studies here presented fifty-six alone are free of this particular foreground study.

### Pictures of Egypt and Life in the Valley of the Nile. Frederick Goodall, R.A. The Fine Art Society.

Representations of rural life are just now to the fore in the Fine Art Society's Galleries. Mrs. Allingham's rural England contrasts strangely with Mr. Frederick Goodall's rural Egypt in neighbouring rooms. Here we find the quaint customs and habits of the Falaheen cleverly portrayed by the artist, whose residence in the land over which the fascination of the "Great Queen" stills reigns supreme have given him much familiarity with his subject. The collection is historical, besides being pictorial. "The" attraction, *par excellence*, of the exhibition, is "The Flight into Egypt," a huge oil which rather dwarfs its surroundings. This is at once a masterpiece of composition and of work. A spirit of reverence pervades the picture more readily felt than described. If alone for this example of his art, Mr. Goodall's exhibition well repays many a visit.

A course of three lectures on "Practical Points for Nurses in Throat, Nose, and Ear Cases," will be delivered under the auspices of the Royal British Nurses' Association, by Mr. R. Lake, at 3, Hanover Square, on the evenings of April 10th, 23rd, and 30th, at eight p.m.

### Everybody's Opinion.

### CO-OPERATION AT BISHOP STORTFORD.

Mrs. Birch, of Linton, Cambridge, writes: As was lately decided, a nurses' co-operation has been opened in Bishop Stortford, as the want of nurses has been keenly felt during the winter months. An appeal was made to the treasurer on behalf of the nurses of the existing Home some time ago, and plans as to the co-operation were laid before him, but no further notice was taken of the matter. Since the opening of the co-operation it has been decided to keep on the original Nursing Home, but there should be scope enough for both institutions to work well. Those who do the work ought certainly to receive the reward of their labours. Why should an institution be called "charitable" if the greater number of its nurses work hard to keep it going, receiving in return only a small remuneration just sufficient for their bare wants. It would be much more truly a "charity" if the subscribers to such institutions would pay and keep their own district nurses, allowing the others to take their own fees minus a small percentage to defray the expenses of the Home. This is what the new co-operation is doing. By and by all the large institutions will probably join to form one great co-operation with branches, then every nurse will receive due payment for her services.

### AN IMPOSTOR NURSE TO BE 'WARE OF, EXPECTED, AND PREPARED FOR.

"A Provincial Matron" writes: I am writing to warn other matrons against an adventuress and thief who is going about the country in the garb of a nurse. She is of medium height, has light auburn hair, a peculiar twitching movement of the eyes, an impediment in her speech, is well educated and ladylike. She presents excellent testimonials, and on the strength of them obtains admittance into Nursing Homes. When she departs, which she generally contrives to do unobserved, she takes with her clothing, jewellery, and money belonging to other people. She has been seen in Douglas, Blackburn, and London, and doubtless institutions in other towns have suffered from her visits.

### ARMY NURSING.

"Nemesis" writes: Your contributor, A. O. Grey, appears to look upon the men of the Medical Staff Corps as "mostly fools," and as I have recently severed my connection with that much abused body of men, I crave permission to say a word in justification of the corps generally. The nursing sister is evidently held in high esteem by the writer of the article in question, and I will not endeavour to combat the laudatory statements. Albeit I should not shirk the task were it imposed upon me. "Their duties are performed in a perfunctory and haphazard manner," but in reviewing the average orderly's life then small wonder will be expressed. He is responsible to the ward master for the cleanliness of his wards, and this entails an amount of rough manual labour that might, and should be performed by an unqualified man. Frequently I have seen a nurse called from scrubbing a floor to attend a case really requiring constant attention, and should an unperformed task of mechanical labour result, woebetide the unfortunate orderly when the surgeon puts in his appearance. The daily instruction class is an institution of very recent date, and I am unable to speak of its use or otherwise; suffice it to say that I am personally acquainted with scores of efficient nurses, men who loved the work and were competent to perform it (and are engaged in nursing work at the present time in civilian life) who have been harassed by petty routine duties until they have fallen into a don't care kind of style, and small wonder either. It is an aspersion on the intellectual capability of the average orderly to say that he does not grasp the idea that a two drachm dose of stimulant administered every hour would not be equivalent to a one drachm dose every half hour, and it is a manifestly unfair statement, unless, indeed, as it appears the writer would have us believe, the men of the Medical Staff Corps are dolts. The qualifications of a successful army sister are also summarised and I would emphasise these, for if all ladies possessed them the life of the orderly would partake less of the toad-under-the-harrow kind of existence that he too frequently has to endure.

### For Reading to the Sick.

**Motto.**

If the best man's faults were written on his forehead, it would make him pull his hat over his eyes.—*Gaelic Proverb.*

**Verses.**

Full sure I am there is no joy in sin;
Sin is established subtly in the heart,
As a disease; like a magician foul,
Ruleth the better thoughts against their will.
Only the rays of God can cure the heart,
Purge it of evil, there's no other way,
Except to turn with the whole heart to God.
—*Allingham.*

Self is earthly—Faith alone
Makes an unseen world our own;
Faith relinquished—how we roam,
Feel our way, and leave our home!
Spurious gems our hopes entice,
While we scorn the pearl of price;
And preferring servant's pay,
Cast the children's bread away.—*Cowper.*

Lovest thou praise? The Cross is shame—
Or ease? The Cross is bitter grief—
More pangs than tongue or heart can frame
Were suffered there without relief. —*Keble.*

Inscribed upon the Cross we see
In shining letters, "God is Love";
He bears our sins upon the Tree,
He brings us mercy from above.
* * * * *
The balm of life, the cure of woe,
The measure and the pledge of love,
The sinner's refuge from below,
The angels' theme in Heaven above.
—*Hymns A. and M.*

**Reading.**

When Thou hidest Thy face they are troubled; when Thou takest away their breath they die. . . . When Thou lettest Thy breath go forth they shall be made, and Thou shalt renew the face of the earth.—*Ps.* civ. 29, 30.

It is not to be doubted that there is much good in a good man, and some good in an evil man, good that God loves to look upon and to help; but there is much evil too, and the danger is lest in cultivating the good we should forget to eradicate the evil. . . . Then again there are a great many persons who have their whole lives in this world embittered, and their whole prospects in the next world ruined, by some secret sin which they love and dare not even tell to God. In fact man, as the Psalmist says, "at his best estate is altogether vanity," and his "iniquities are more in number than the hairs of his head." Lord have mercy, should be our daily prayer; for as St. John says, "If we say that we have no sin we deceive ourselves, and the truth is not in us; but if we confess our sins, He is faithful and just to forgive us our sins, and to cleanse us from all unrighteousness."—*Rev. E. Adams.*

"The world," says St. John, "is passing away and the lust thereof, but he that doeth the will of God abideth for ever. . . . We shall not be faultless in the future; but we may do better than we have done, and then better and better still. . . . Only let us look right up to Him; right away from ourselves, chastened and sobered by the past, but not degraded or despondent; dead indeed unto sin, but alive unto God—alive with His own life of love, "Who being raised from the dead dieth no more."

### The Book World for Women and Nurses.

[We invite Correspondence, Criticism, Enquiries, and Notes on Books likely to interest Women and Nurses. Address, Editor, The Hospital (Nurses' Book World), 428, Strand, W.C.]

A Yellow Aster. By Iota. (Hutchinson and Co. 1894.)

Following closely on the lines of various other popular romancers of the same order, the authoress apparently has no literary ambition other than in playing to the gallery. The attempt at realism which pervades "Iota's" volumes has none of the great atoning virtues of that school. The writer of "A Yellow Aster" has, in fact, all the faults and none of the virtues of realism. She is literal without being literary. Suggestion never contents her. There may be other readings, but the plot shows us the self-analysing, self-centred girl Gwen, evolving through volumes I, II, and III into the "woman" Gwen. Her development into rational womanhood is, indeed, the motif of "Iota's" present masterpiece. To get at this final consummation, after come the baronet husband and the baby; but she does not love either readily. Page upon page of hysterical morbid introspection on the part of the heroine (she is a great beauty, of course) wind us up to a pitch where double murder, to say the least, is to be anticipated. But lo! we leave "The Yellow Aster" submissive, repentant, loving, prostrate at the feet of her Humphrey. Up to this point Gwen's struggles against the love which lies dormant in her nature are described in an exhaustive manner, until, as our authoress tersely puts it, her heroine's control over herself was on its "last legs.' But, after all, one asks oneself is "A Yellow Aster" meant to be taken seriously?

"Do You Know It? If Not You Should." (Saxon and Co. London, 1894.)

We recommend everyone to become possessed of a copy of the above little book. It is readily obtained at all booksellers, and is to be found on the Metropolitan book stalls. Thus for the modest outlay of 6d. one can become possessed of pleasing knowledge of words and phrases which one hears and uses every day and yet which one applies without a corresponding understanding. In 1894 we are all political, superficially it may be, but we all have our views and our parties. To be allies in a more real sense of the word we should understand the grammar of our politics. Many of us—far too many—are herein deficient. This small volume puts it into the power of all to be more intelligent in the use of our tools, better able both to speak and to understand. The selection of words which Mr. C. E. Clark incorporates in his book have been made from the standpoint of the general reader, "because," as the Editor remarks in his preface, "he can scarcely take up a newspaper, a review, or a publication of any kind in which he does not meet with expressions with often an inadequate recognition of their meaning." We wish the compiler all success with his excellent little book.

Theories: or Studies from a Modern Woman. A. N. T. A. P. (London: T. Fisher Unwin. 1894.)

This small work, which is essentially by a modern woman, distinctly shows us that theories, however overdone and boring in themselves, can form, if judiciously put into separate characters, a story which is both clever and interesting. It is a tale of a hysterical young woman who has based her life on certain theories, and possesses some curious notions on love, marriage, religion, socialism, and the education of her children. In fact, at the age of 19, Beatrice Marlone considers she has almost solved life's problems, and then commences to work out her extensive knowledge of men and things. In hopes of gaining more liberty to advance her ideas, she consents to an engagement and marriage with Frank Lufmoor, a wealthy, kind-hearted, but very ordinary young country squire, who is devoted to her. It is to Marion Hauth, her greatest friend—who is a very well-drawn specimen of the contented, common-place woman, who of course could not but fail to understand her—that Beatrice eloquently expresses her horror and contempt at the idea of being in love. "In love! really Marion you are too bad; why, I have said over and over again, that it is simply impossible for educated, refined, cultivated people even to fall in love now. It is a conventional term simply for an out-of-date emotion. . . . . What is the use of being highly civilised if one is to retain all the passions of a primitive time." To start with such ideas, and to pursue the workings of the same, was hardly conducive to a happy married life, especially with such a husband as Frank Lufmoor; though he is oft-times long suffering enough, yet could never hope to follow her height—even if misguided—of thought. We can feel and admire both husband and wife. It is marvellous how she maintains her enthusiasm, and perseveres after each pet hobby falls to the ground, and will not recognise failure until the entire relationship between herself and her children prove ruinous to both; then at last does her spirit give way, and she is humiliated into a rude awakening. At this point a true realistic touch of nature is brought in, and we are sorry in one way when the excitable, theoretical woman relapses into a commonplace wife and mother.

Prior Rahere's Rose. A Story of St. Bartholomew's, Smithfield. (Griffith, Farran, and Co.)

Many interesting traditions and connecting links with past ages are associated with buildings familiar but representing little more to us than brick and stone, and the present purpose they serve. No hospital in London has a more interesting chronicle than old St. Bartholomew's. Yet its history is but little known. Some small part of it must become known to all who penetrate within the building, as one ward still bears the name of Rahere, that Prior of the monastry of St. Bartholomew to whom the founding of the hospital is due. St. Bartholomew's Hospital was the outcome of a vision. Those who do not know the story, or only know part of it, I would refer to the charming chronicles collected by Miss Channing, and published in the little book called "Prior Rahere's Rose."

### THE MAGAZINES.

The Fortnightly Review for April contains an interesting paper on "Fly-fishing," by Basil Field, which will be of much acceptance to him "who casts his line in solitude." Here we find an exhaustive investigation of statistics regarding the growth of the fly-fisher, and a review of the literature concerning his art. Mr. T. W. Russell's paper contributes some terse comments on "The Government and the Evicted Tenants." But the contribution *par excellence* to this issue is Mr. W. D. Morrison's on the subject of our prison system. "Are our Prisons a Failure?" he asks, and goes on to point out much to prove this unhappy truth. "What is the meaning of this ever-increasing outlay on the repression of crime?" This is a question which the public has a right to ask. It is a question which everyone connected with the criminal administration is bound by obligations of duty to answer. I am free to confess that a conviction has been growing up in the minds of some of us who work in prisons that the vast sums the country has now to pay on account of crime, and the vast increase which has taken place in the numbers of the police, are partly due to the growing inefficiency of our prison system as a deterrent force. . . . Deterrence is the principle by which our prisons must ultimately be judged, and if our English prisons are judged by this principle, Mr. Morrison points out, "we shall have to reconsider several of our ideas respecting their boasted superiority."

## Figures and Tables

**Figure f1:**